# Urinary Concentrations of Triclosan, Bisphenol A, and Brominated Flame Retardants and the Association of Triclosan with Demographic Characteristics and Body Fatness among Women with Newly Diagnosed Breast Cancer

**DOI:** 10.3390/ijerph19084681

**Published:** 2022-04-13

**Authors:** Mmadili N. Ilozumba, Weilin L. Shelver, Chi-Chen Hong, Christine B. Ambrosone, Ting-Yuan David Cheng

**Affiliations:** 1 Department of Epidemiology, College of Public Health and Health Professions and College of Medicine, University of Florida, Gainesville, FL 32610, USA; 2 Biosciences Research Laboratory, USDA-ARS Edward T. Schafer Agricultural Research Center, Fargo, ND 58102, USA; weilin.shelver@usda.gov; 3 Department of Cancer Prevention and Control, Roswell Park Comprehensive Cancer Center, Buffalo, NY 14203, USA; chi-chen.hong@roswellpark.org (C.-C.H.); christine.ambrosone@roswellpark.org (C.B.A.)

**Keywords:** triclosan, bisphenol A, breast cancer, menopausal status, body size, body composition

## Abstract

Background: Triclosan, bisphenol A (BPA), and brominated flame retardants are environmental estrogenic endocrine-disrupting compounds that may influence the prognosis of breast cancer. We examined the urinary concentrations of these compounds and their associations with demographic characteristics and body fatness in a population of women with newly diagnosed breast cancer. Methods: Overnight urine collection and anthropometric measures were obtained from 302 participants. Triclosan, BPA, tetrabromobisphenol A (TBBPA), and tetrabromobenzoic acid (TBBA) concentrations were determined using ultra-performance liquid chromatography–tandem mass spectrometry. Regression analyses were conducted to examine associations of urinary compound concentration with age, menopause, race, ethnicity, educational level, estrogen receptor status, body size, and body composition. Results: Triclosan, BPA, and TBBA were detected in urine samples from 98.3%, 6.0%, and 0.3% of patients, respectively; TBBPA was undetectable. Among patients with quantifiable values, the geometric mean concentrations were 20.74 µg/L (27.04 µg/g creatinine) for triclosan and 0.82 µg/L (1.08 µg/g creatinine) for BPA. Body mass index ≥ 30 vs. <25 kg/m^2^ was associated with lower creatinine-corrected urinary concentrations of triclosan (−40.00, 95% confidence interval [CI] = −77.19 to −2.81; *p* = 0.0351). The observed association was predominantly in postmenopausal women (−66.57; 95% CI: −109.18% to −23.96%). Consistent results were found for associations between triclosan levels and fat mass variables. Conclusion: In this study population, women with newly diagnosed breast cancer had triclosan exposure. Assessments of the implications of urinary concentrations of triclosan for women should consider body fatness and menopausal status.

## 1. Introduction

Triclosan, bisphenol A (BPA), and brominated flame retardants are endocrine-disrupting compounds (EDCs) that are commonly present in the environment [1,2]. The estrogenic effects of these EDCs may result in DNA damage and variants of oncogenes and tumor suppressor genes, as well as changes in gene expression in breast epithelial cells [3,4]. These compounds are present in cosmetics, personal care products, plastics, and the environment [2]. Triclosan is used as an antibacterial agent in personal care products such as soaps, deodorants, toothpastes, laundry detergents, and disinfection solutions, and as a material preservative [5,6]. BPA is predominantly found in polycarbonate plastics, such as drink containers, canned foods and beverages, toys, bottles, water pipes, and medical equipment [2,7,8]. Brominated flame retardants, for instance, tetrabromobisphenol A (TBBPA) and 2-ehtylhexyl-2,3,4,5-tetrabromobenzoate (TBB), are chemicals added to products such as plastics, textiles, furniture, electronics, home appliances, and industrial equipment [9]. Despite governmental restrictions in the use of some of these EDCs in consumer products, they may be ubiquitous and remain persistent in the environment, thereby easily contaminating food and water. Although dietary intake may be a primary route of exposure to these EDCs in the general population, air inhalation and skin absorption are also well known routes of exposure [6,9,10]. These compounds have been detected in urine, breast milk, blood, hair, and adipose tissue samples [1,11,12,13,14].

Demographic factors, such as sex and body mass index (BMI), are potentially important determinants of urinary concentrations of EDCs [14,15,16,17,18]. For triclosan, the National Health and Nutrition Examination Survey (NHANES) 2003–2010, a large representative sample of the U.S. general population, showed that women had higher urinary concentrations (geometric mean = 29.3 μg/g creatinine, 95% confidence interval [CI] = 26.6–32.1) than men (geometric mean = 26.2 μg/g creatinine, 95% CI = 23.7–28.9) [14]. Li and colleagues reported an inverse association between urinary triclosan concentrations and BMI and waist circumference (WC) in the NHANES data [14]. The reason for the inverse association is unclear, as EDCs are associated with increased risk in obesity [19]. A potential mechanism is that a portion of EDCs can be sequestered in adipose tissue [20], leading to an inverse association in cross-sectional studies. To further understand the mechanism, it is important to explore the urinary concentrations of EDCs in association with body composition, providing a different aspect of assessment of body fat in addition to BMI and WC.

Biomonitoring and epidemiological studies have examined levels of these EDCs among children and adult populations in different countries [14,16,18,21,22,23,24,25,26,27]. For women, levels have been examined mostly among pregnant women and women with fertility issues undergoing in vitro fertilization [21,28,29]. However, there is limited information on the urinary concentrations of EDCs in women who have received a diagnosis of breast cancer, a population that may be susceptible to the adverse effects of EDCs, including hormonal imbalance and poor cancer prognosis [1,3,4,30]. Here, we report on urinary concentrations of triclosan, BPA, TBBPA, and tetrabromobenzoic acid (TBBA), a metabolite of TBB, in women newly diagnosed with breast cancer. We also investigated the extent to which demographic characteristics, body size, and body composition are associated with urinary concentrations of these EDCs.

## 2. Methods

### 2.1. Study Population

Patients with breast cancer included in this study were participants in the Women’s Health after Breast Cancer (ABC) Study, a hospital-based, prospective cohort study that included women with incident breast cancer who were treated at Roswell Park Comprehensive Cancer Center and were initially enrolled in the center’s Data Bank and BioRepository (DBBR). Detailed methods of the DBBR and the ABC Study have been published elsewhere [31,32]. Briefly, 423 women with early-stage (0 to IIIa), non-metastatic breast cancer were recruited between 17 March 2006 and 22 April 2010. The initial goal of the study was to examine the determinants of weight gain after breast cancer diagnosis. As part of the DBBR protocol, a set of standardized questionnaires was administered at diagnosis to collect information on demographic characteristics, lifestyle factors, dietary intake, and the use of supplemental vitamins and prescription and non-prescription drugs. Clinical data on estrogen receptor status was abstracted from pathology reports. Menopause was defined as self-reported cessation of menses in the past year, either as natural menopause or due to hysterectomy with bilateral oophorectomy. Overnight urine samples were collected on the morning of surgery at the time of diagnosis. Participants were instructed to void the bladder just before going to bed in the evening or at 11 p.m. and to collect all urine passed overnight until the first void in the morning. Urine samples were obtained within 4 h of collection, aliquoted as unfractionated samples with HCl as a preservative, and stored in −80 °C freezers until analysis. Written informed consent was obtained from all participants. The study was approved by the Institutional Review Board at Roswell Park Comprehensive Cancer Center.

### 2.2. Anthropometric and Body Composition Measurements

Anthropometric measurements were obtained by trained staff during in-person interviews using a standardized protocol described elsewhere [33]. Participants were asked to wear light clothing and to remove their shoes and heavy jewelry. Waist and hip circumferences were measured by placing the measuring tape around the waist covering the umbilicus for the waist and at the maximum extension of the buttocks in a horizontal plane for the hip. The waist and hip measurements were recorded to the nearest 0.1 cm, and the waist-to-hip ratio was calculated as the waist circumference divided by the hip circumference. Standing height was measured once to the nearest 0.1 cm. Body composition was measured by bioelectrical impedance analysis using a Tanita^®^ TBF-300A scale, and the data were transformed to fat mass in kg, fat mass index, percentage body fat, percentage trunk fat, and trunk fat mass. Weight was measured once using the Tanita scale. BMI was calculated as body weight in kg divided by height in meters squared. The fat mass index was calculated as fat mass in kg divided by height in meters squared.

### 2.3. Laboratory Analysis

Laboratory analyses of the EDCs were performed at the U.S. Department of Agriculture, Agriculture Research Service, Biosciences Research Laboratory. Urine creatinine concentrations were measured with a Vitros Fusion 5.1 clinical chemistry analyzer (Ortho Clinical Diagnostics) using a slide method at the Clinical Laboratories at Roswell Park Comprehensive Cancer Center.

### 2.4. Materials

Liqua-Trol, a control for human urinalysis, was obtained from KOVA International Inc. (Garden Grove, CA, USA). Standards for 2,3,4,5-TBBA, 2-ethylhexyl 2,3,4,5-Tetrabromobenzoate (TBB), and di(2-ethylhexyl) tetrabromophthalate were purchased from AccuStandard, Inc. (New Haven, CT, USA). BPA-*d*_6_ and triclosan-*d*_3_ were obtained from Toronto Research Chemicals Inc. (Toronto, ON, Canada). BPA, TBBPA, and 2,3,5-triiodobenzoic acid (TIBA) were purchased from Sigma-Aldrich (St. Louis, MO, USA). TBBA-^13^C_12_ was obtained from Cambridge Isotope Laboratories, Inc. (Tewksbury, MA, USA). Triclosan was purchased from United States Pharmacopeia (Rockville, MD, USA).

### 2.5. Sample Preparation

Control urine or collected urine samples (2 mL) were mixed with 100 µL of 20 ng/mL TIBA, deuterated or ^13^C-labeled compounds, and 200 µL of 10 N NaOH, and the mixture was heated at 100 °C for 90 min. For the calibration curve, 100 µL of calibration standards of 2, 10, 20, 100, 200, and 400 ng/mL were added to control urine to generate a matrix-matched calibration curve. An alkaline hydrolysis step was used to hydrolyze the glucuronide conjugates of the phenolic compounds or hydrolyze esters. The hydrolysis efficiency was determined by hydrolyzing 25 ng/mL of TBB into TBBA in control urine and found to be at 66.4 ± 5.4% (*n* = 27). After alkaline hydrolysis, samples were cooled for 30 min, and if needed, deionized water was added to bring the sample to the original weight, followed by the addition of 300 µL of 6 N HCl. The solution was mixed with 2.5 mL of acetonitrile and incubated for 30 min. Ethyl acetate (4 mL) was used to extract the urine solution, and the extraction process was repeated three times. The combined ethyl acetate extracts were evaporated with a stream of nitrogen to dryness. The vial was then reconstituted with 200 µL of MeOH, and the solution was stored at −20 °C in a liquid chromatography–mass spectrometry (LC-MS) vial, with a silanized vial insert until analyzed.

### 2.6. LC-MS/MS Analysis

Sample analysis was conducted using ultra-performance (UP) LC-tandem mass spectrometry (MS/MS) with equipment consisting of a Waters Acquity UPLC system, in conjunction with a Waters triple quadrupole mass spectrometer. Sample aliquots (10 µL) were injected onto an Acquity UPLC™ HSS T3 column (1.8 µm, 2.1 × 100 mm; Waters, Milford, MA, USA) equipped with a VanGuard precolumn (1.8 µm, 2.1 × 5 mm). The autosampler was maintained at 5 °C and the column at 30 °C. The binary gradient system consisted of solvent A (5 mm ammonium acetate) and solvent B (MeOH). The solvent flow rate was 0.3 mL/min, and the program was set for 0–0.5 min, 10% B → 50% B; 0.5–5.5 min, 50% B → 100% B; 5.5–9.5 min, isocratic at 100% B; 9.5–10 min, 100% B → 10% B; and 10–15 min, 10% B.

Data were acquired, processed, and quantified using MassLynx^TM^ 4.1 with TargetLynx^TM^ software (Waters Corporation, Milford, MA, USA). Mass spectrometric conditions for TBBA, TIBA, BPA, TBBPA, triclosan, or BPA-*d*_6_, triclosan-*d*_3_, TBBA-^13^C_12_ were optimized by direct infusion using electrospray ionization in the negative mode to identify the precursor ion, product ions, and the optimum collision energies and cone voltage using AutoTune Wizard with MassLynx^TM^ 4.1 software. The desolvation temperature was 500 °C, and the source temperature was 120 °C. Nitrogen used as the cone gas was set at 50 L/h, the desolvation gas flow was 800 L/h, and the collision gas flow of argon was 0.17 mL/min. Ions were monitored in the multiple reaction monitoring mode. The quantitation was based on *m*/*z* 436.5 > 392.3 for TBBA, 498.6 > 454.3 for TIBA, 227.0 > 211.7 for BPA, 288.8 > 34.8 for triclosan, and 542.5 > 290.6 for TBBPA. The qualification was based on *m*/*z* 436.5 > 78.8 for TBBA, 498.6 > 126.6 for TIBA, 277.0 > 133.1 for BPA, 288.8 > 36.8 for triclosan, and 542.5 > 446.3 for TBBPA. The limits of detection were calculated based on t_0.99_ times the standard deviation of eight analyses and were 0.42 ng/mL for TBBA, 0.45 ng/mL for BPA, and 2.2 ng/mL for triclosan. The limits of quantitation were calculated based on three times the limit of detection and were 1.4 ng/mL for TBBA, 1.5 ng/mL for BPA, and 6.5 ng/mL for triclosan. If sample concentrations were above the limits of quantitation, but the ion ratios did not meet, the estimated maximum possible concentrations were reported. Unknown concentrations were determined by LC-MS/MS using a matrix-matched standard curve and TIBA, BPA-*d*_6_, triclosan-*d*_3_, and TBBPA-^13^C_12_ as the internal standards using linear calibration with 1/x weighting. For regression analyses, urinary concentrations were adjusted for creatinine using the covariate-adjusted standardization procedure [34]. Log-transformed creatinine was first regressed on covariates including age, BMI, menopausal status, race, and education. Observed creatinine concentrations were then divided by the fitted creatinine values obtained from this model, producing a ratio representing the covariate-independent residual effect of hydration on creatinine. Then, we standardized urinary triclosan concentrations by dividing the biomarker concentrations by the ratio of observed to fitted creatinine.

### 2.7. Statistical Analysis

Data from 302 patients with urinary measurements of EDCs and creatinine were included in the analyses. Independent variables were categorized for age (<50, 50–59, 60–69, and ≥70 years), menopause (no, yes), race (White and all other races), educational level (grade school or some high school, or high school graduate or equivalent, some college, college graduate, and advanced degree), estrogen receptor status (positive, negative), and BMI (<25, 25–30, and ≥30 kg/m^2^), whereas other body size and body composition measurements were categorized as quartiles. Generalized linear model analysis was used for the creatinine-corrected triclosan concentrations in relation to the independent variables. Body size and composition measurements were independently included in the model to avoid multicollinearity. Because BPA, TBBA, and TBBPA were detected in a small percentage of samples or not detected, no regression analysis was performed for these compounds. For the associations of body size and body composition with triclosan concentrations, we conducted analyses stratified by premenopausal and postmenopausal status because women’s body fatness and composition may change substantially after menopause. The Wald test of the two-way interaction between body size and body composition variables and menopausal status was used to evaluate statistical effect modifications. All statistical tests were two-sided, and statistical significance was *p* < 0.05. Analyses were performed using SAS, version 9.3 (SAS Institute Inc., Cary, NC, USA).

## 3. Results

Baseline demographic and clinical characteristics of the 302 study participants included in the analyses are given in Table 1. One-third of participants were 50 years or younger (32.1%), and another third were 50–59 years of age (32.8%). The majority of women were White (92.8%), and had some college or higher education (70.4%). Two-thirds of the women (63.8%) were postmenopausal, and one-third (39.1%) were obese (i.e., BMI ≥ 30 kg/m^2^). Among patients with known tumor receptor status, 78.6% had estrogen receptor-positive tumors.

Urine EDC levels are given in Table 2. The geometric mean of the urine triclosan concentration was 20.74 µg/L (27.04 µg/g creatinine). Urine BPA was detected in 18 participants (6.0%), and the geometric mean of urine BPA was 0.82 µg/L (1.08 µg/g creatinine). Urine TBBPA was undetected in all participants. Urine TBBA was detected in one participant.

We observed an inverse borderline association that was slightly above our cutoff for statistical significance between race and creatinine-corrected concentrations of triclosan in the unadjusted model (Table 3). Women of all other races vs. White women were associated with lower concentrations of creatinine-corrected urinary triclosan (−49.03, 95% CI = −106.45, 8.39, *p* = 0.0939). Age range between 50 to 59 years vs. less than 50 years was associated with lower concentrations of creatinine-corrected urinary triclosan (−44.19, 95% CI = −80.40, −7.97, *p* = 0.0170). College graduate vs. grade school/some high school and high school graduate/GED was associated with a borderline increased concentrations of creatinine-corrected urinary triclosan (35.41, 95% CI = −7.79, 78.60, *p* = 0.0953). By contrast, we observed no associations among individual demographic characteristics with creatinine-corrected urinary triclosan concentration after covariate adjustment. However, BMI was inversely associated with urine triclosan concentrations (Table 4). Obese women vs. normal-weight women were associated with lower concentrations of creatinine-corrected urinary triclosan (−40.00, 95% CI = −77.19% to −2.81%; *p* = 0.0351). In general, higher vs. lower levels of central adiposity and fat mass amount and percentages were associated with lower concentrations of creatinine-corrected triclosan. However, these associations were not statistically significant.

Table 5 provides the results of the analyses stratified by menopausal status for the associations of body size and body composition with creatinine-corrected triclosan concentration. Menopausal status was not a significant effect modifier for the association between BMI and creatinine-corrected triclosan. However, in the stratified analysis, the association was statistically significant for postmenopausal women (−66.57, 95% CI = −109.18, −23.96 for obese vs. normal-weight groups), but not for premenopausal women. In contrast, menopausal status was a significant effect modifier of the association between waist-to-hip ratio and creatinine-corrected triclosan (P-interaction = 0.0348) and the associations between body composition measurements and creatinine-corrected triclosan (P-interaction: 0.0050 for percentage body fat, 0.0175 for fat mass, 0.0011 for percentage trunk fat, and 0.0037 for trunk fat mass). Although inverse associations were observed for postmenopausal women, there was no association, or a slight indication of a positive association, for premenopausal women.

## 4. Discussion

The present study is among the few to examine the urinary concentrations of EDCs and their associations with demographic factors and body fatness among women with newly diagnosed breast cancer. Of the EDCs we assayed in urine samples, triclosan was detected in most participants, whereas BPA was detected in <10% of participants. TBBA and TBBPA were essentially undetected in our samples.

Our study participants had urinary triclosan concentrations (geometric mean, 27.0 μg/g creatinine) similar to those for women in the 2003–2010 NHANES study (geometric mean, 29.3 μg/g creatinine) [14]. In our study population, obesity (BMI ≥ 30) was associated with lower creatinine-corrected urinary triclosan concentrations. Consistent with this result, the NHANES 2003–2010 study found an inverse association between urinary triclosan concentrations and BMI and WC [14]. However, to our knowledge, our study is the first to report more comprehensive adiposity measurements and their associations with urinary triclosan concentrations. The results for WC, waist-to-hip ratio, and fat mass were similar to those for BMI, suggesting that lower urinary triclosan concentrations were associated not only with central adiposity, but also with adiposity in general, with muscle and other body components less likely to be involved in the associations. The biological mechanisms undergirding this finding are unclear because the observations are inconsistent with prior research showing that EDC exposure at a window of susceptibility alters endocrine regulation, thereby increasing obesity risk [21,35,36]. Specifically, EDCs may alter energy metabolism and hormonal control of adipose tissue functions, which may induce inappropriate deposits of fat leading to obesity [20,24,36,37]. On the other hand, because triclosan and some other EDCs can be sequestered in adipose tissue [20], it is plausible that lower urinary concentrations of EDCs in individuals with obesity indicate greater sequestration of these chemicals in adipose tissue. Using urinary concentrations of triclosan for exposure assessment may need to be reassessed for such individuals. However, previous research has shown that urinary and adipose tissue triclosan levels are not correlated [37], suggesting that concentrations in adipose tissue and urine may indicate different metabolic pathways.

In our study, BPA was detected in <10% of individuals, and the brominated flame retardant TBBPA and the brominated flame retardant metabolite TBBA were essentially undetected. These observations are not entirely consistent with national data. In the 2003–2004 NHANES data, BPA was detected in most women, with a geometric mean of 2.4 µg/L [38]. In the 2013–2014 NHANES data, the concentrations were 1.15 µg/L and 1.36 µg/g creatinine [39], suggesting that BPA levels have declined over time in the U.S. All these values are higher than those detected in our population (BPA, 1.08 µg/L). In a case-control study that included patients with breast cancer, the Long Island Breast Cancer Study Project, BPA was detected in 82% of 893 participants, with median concentrations of 1.20 µg/L and 1.53 µg/g creatinine in breast cancer cases compared with 1.30 µg/L and 1.69 µg/g creatinine in controls [40]. The proportion of participants with detectable BPA concentrations in urine in that study was much larger than in our study participants, and the median concentrations were higher. The urine samples in the Long Island study were collected in 1996 and 1997, which was approximately 10 years earlier than our study participants’ urine collection (2006–2010), and was prior to when manufacturers largely phased out using BPA plastics for containers or water bottles. Notably, other bisphenols are gradually replacing BPA in industrial products, which may explain the presence of BPA in <10% of our study samples. For TBBA, a high percentage (76.9%) of detectable concentrations was reported among men and women who were likely to have occupational exposure [41]. In the 2013–2014 NHANES data, however, TBBA was detected in <5% of men (geometric mean = 0.06 μg/L and 0.143 μg/g creatinine) and was essentially undetectable in women [39]. This may explain the undetectability of TBBA and TBBPA in our study, as our participants were women who were, for the most part, unlikely to have occupational TBBPA exposure. TBB was introduced as a replacement for pentabrominated diphenyl ether (PBDE). Because the banning of PBDE took effect around 2007, the prevalence of TBB and its metabolite TBBA in human urine may not be as high in our study as it would be at a later date [30,32]. Another source of variation in urinary concentrations is that BPA and some brominated flame retardants, such as TBBPA and hexabromocyclododecane, have short biological and elimination half-lives, and thus urine measurements most likely indicate recent exposures [19,42,43,44].

Menopausal status was a significant effect modifier for the associations between urinary triclosan concentration and waist-to-hip ratio, percentage body fat, fat mass, percentage trunk fat, and trunk fat mass. In the analysis stratified by menopausal status, an inverse association was generally found among postmenopausal women, whereas no association or an indication suggestive of a positive association was found in premenopausal women, which suggests intrinsic biological differences in adiposity or metabolism due to menopause. Compared with premenopausal women, postmenopausal women have more fat than muscle [45,46], which may explain why there was a stronger inverse association among postmenopausal women. There are very limited data on triclosan distribution stratified by menopausal status, and our findings warrant further confirmation.

One of the strengths of our study was that overnight urine samples were used, providing less hour-to-hour variation compared with spot urine samples. The urine was collected at the time of surgery; thus, the concentrations of the measured compounds were less likely affected by breast cancer treatments, such as chemotherapy and radiation, and lifestyle changes after breast cancer diagnosis. Two additional strengths of our study were the more comprehensive measurement of body fatness given the potential for misclassification of adiposity using BMI, and the collection of anthropometric data by trained staff with a standardized protocol.

A major limitation of our study is its cross-sectional nature. We were unable to determine the temporality of body fatness and triclosan exposure. Some of the demographic factors were based on self-reporting. Women may be unable to accurately categorize menopause, especially during the perimenopausal period. Residual confounding may also be an issue, despite controlling for some important confounders in the study. We analyzed only one urine sample per patient at the time of breast cancer diagnosis. Nevertheless, a single urine sample may suffice as a measure of longer-term EDC exposure because the measure of reproducibility, i.e., intra-individual variability, reported for these compounds among pregnant women is fair to good [47,48]. Not determining the concentration of EDCs in adipose tissue may hamper the interpretation of findings based on urinary measurements. The generalizability of this study may be limited because the participants were women with a breast cancer diagnosis. Another limitation of our study is that we did not consider other BPA analogues that may have a disruptive effect on the endocrine system comparable to that of BPA. BPA is subject to many restrictions and therefore, less and less present on the market. It is therefore important to consider BPA analogues replacing the use of BPA in future studies.

## 5. Conclusions

In a study population of women with newly diagnosed breast cancer, triclosan was the most detectable EDC measured. BMI was inversely associated with urinary triclosan concentrations, particularly in postmenopausal patients. The exposure assessment using the urinary concentrations of triclosan should take into account body fatness and menopausal status. Given that body fatness plays an important role in breast cancer prognosis and that triclosan has potential effects in cancer development, prospective studies are needed to delineate the underlying mechanisms and implications of these two factors for women with breast cancer.

## Figures and Tables

**Table 1:** Demographic and clinical characteristics of 302 included study patients.

Characteristic	No.	Col %
Age (years)		
<50	97	32.1
50–59	99	32.8
60–69	74	24.5
≥70	32	10.6
Menopause status		
Premenopausal	106	36.2
Postmenopausal	187	63.8
Missing	9	
Race		
White	272	92.8
All others	21	7.2
Missing	9	
Educational level		
Grade school/some high school/high school graduate/GED	86	29.6
Some college	96	33.0
College graduate (4 years)	56	19.2
Advanced degree	53	18.2
Missing	11	
Tumor ER Status		
ER positive	220	78.6
ER negative	60	21.4
Body mass index, kg/m^2^		
<25	89	29.5
25 to <30	95	31.5
≥30	118	39.1
Percentage body fat		
≤35.00	63	25.3
>35.00 to ≤41.10	63	25.3
>41.10 to ≤45.50	61	24.5
>45.50	62	24.9
Missing	53	
Fat mass, kg		25.3
≤22.70	63	24.9
>22.70 to ≤30.80	62	24.9
>30.80 to ≤39.20	62	24.9
>39.20	62	25.3
Missing	44	
Fat mass index, kg/m^2^		
≤8.74	63	25.3
>8.74 to ≤11.64	61	24.5
>11.64 to ≤14.75	62	24.9
>14.75	63	25.3
Missing	44	
Waist circumference, inches		
≤79.00	67	25.8
>79.00 to ≤89.00	69	26.5
>89.00 to ≤101.40	59	22.7
>101.40	65	25.0
Missing	33	
Waist to hip ratio		
≤0.78	69	26.6
>0.78 to ≤0.83	65	25.1
>0.83 to ≤0.87	58	22.4
>0.87	67	25.9
Missing	34	
Percentage trunk fat		
≤32.60	63	25.3
>32.60 to ≤39.10	62	24.9
>39.10 to ≤44.40	62	24.9
>44.40	62	24.9
Missing	44	
Trunk fat mass, kg		
≤12.00	63	25.3
>12.00 to ≤16.40	65	26.1
>16.40 to ≤21.00	59	23.7
>21.00	62	24.9
Missing	44	

Col indicates column; ER, estrogen receptor; GED, General Education Development (degree); No., number.

**Table 2:** Concentrations of endocrine-disrupting compounds detected in the urine of 302 women with newly diagnosed breast cancer.

Endocrine-Disrupting Compound	No. of Samples with Detected Concentrations (% of total)	Concentration, μg/L			Concentration, μg/g Urine Creatinine		
		Mean ± SD	Geometric Mean	Range (Minimum–Maximum)	Mean ± SD	Geometric Mean	Range (Minimum–Maximum)
Triclosan	297 (98.3)	68.31 ± 134.81	20.74	0.11–974.86	88.74 ± 178.98	27.04	0.19–1346.91
Bisphenol A	18 (6.0)	1.79 ± 3.91	0.82	0.13–17.27	2.80 ± 6.22	1.08	0.17–27.17
Tetrabromobisphenol A	0 (0)	NA	NA	NA	NA	NA	NA
Tetrabromobenzoic acid	1 (0.3)	0.31 ± (−)	0.31	0.31–0.31	0.79 ± (−)	0.79	0.79–0.79

NA indicates not applicable; SD, standard deviation. Urine creatinine = concentration of endocrine disrupting compound divided by urine creatinine.

**Table 3:** Associations of demographic variables with urinary creatinine-corrected triclosan concentration.

Variable		Unadjusted			Adjusted ^a^	
	*n*	Beta Estimate (95% CI)	*p*-Value	*n*	Beta Estimate (95% CI)	*p*-Value
Age (years)	291			291		
<50		Ref.			Ref.	
50–59		−44.19 (−80.40, −7.97)	0.0170		−24.75 (−71.87, 22.36)	0.3019
60–69		−18.47 (−57.76, 20.81)	0.3554		6.67 (−49.98, 63.32)	0.8169
≥70		−6.30 (−57.33, 44.73)	0.8083		11.84 (−54.06, 77.73)	0.7239
Menopausal Status	291			291		
Premenopausal		Ref.			Ref.	
Postmenopausal		−24.41 (−54.62, 5.79)	0.1127		−12.41 (−59.02, 34.21)	0.6007
Race	291			291		
White		Ref.			Ref.	
All others		−49.03 (−106.45, 8.39)	0.0939		−43.93 (−101.19, 13.32)	0.1321
Educational level	291			291		
Grade school/some high school/high school graduate/GED		Ref.			Ref.	
Some college		−2.10 (−38.83, 34.62)	0.9103		−9.56 (−46.92, 27.79)	0.6147
College graduate (4 years)		36.12 (−6.35, 78.60)	0.0953		25.67 (−17.53, 68.87)	0.2431
Advanced degree		35.41 (−7.79, 78.60)	0.1078		23.19 (−20.54, 66.91)	0.2974
Tumor ER Status	278			278		
Positive		Ref.			Ref.	
Negative		−12.37 (−47.85, 23.10)	0.4929		−12.01 (−47.55, 23.54)	0.5066

CI indicates confidence interval; ER, estrogen receptor; GED, General Education Development (degree); and Ref., reference. ^a^ Multivariable analyses were adjusted for age (<50, 50–59, 60–69, ≥70 years), body mass index (<25, 25–30, ≥30 kg/m^2^), menopause (no, yes), race (White, all others), and educational level (grade school/some high school/high school graduate or equivalent, some college, college graduate, advanced degree). Urinary creatinine-correction = covariate-adjusted standardization.

**Table 4:** Associations of body size and body composition measures with urinary creatinine-corrected triclosan concentrations.

Variable		Unadjusted			Adjusted ^a^	
	*n*	Beta Estimate (95% CI)	*p*-Value	*n*	Beta Estimate (95% CI)	*p*-Value
BMI, kg/m^2^	291			291		
<25		Ref.			Ref.	
25 to <30		−21.00 (−58.27, 16.27)	0.2683		−13.48 (−51.12, 24.16)	0.4814
≥30		−53.33 (−89.03, −17.64)	0.0035		−40.00 (−77.19, −2.81)	0.0351
Waist circumference, inches	258			247		
≤79.00		Ref.			Ref.	
>79.00 to ≤89.00		15.59 (−25.66, 56.83)	0.4575		26.61 (−15.10, 68.32)	0.2101
>89.00 to ≤101.40		−48.05 (−91.18, −4.92)	0.0291		−40.67 (−84.78, 3.44)	0.0706
>101.40		−42.47 (−84.50, −0.43)	0.0477		−22.54 (−66.62, 21.54)	0.3149
Waist-to-hip ratio	257			246		
≤0.78		Ref.			Ref.	
>0.78 to ≤0.83		−27.24 (−68.64, 14.16)	0.1963		−21.08 (−63.44, 21.28)	0.3279
>0.83 to ≤0.87		−20.54 (−63.20, 22.13)	0.3441		−12.48 (−55.64, 30.68)	0.5695
>0.87		−24.19 (−65.59, 17.21)	0.2509		−12.85 (−55.89, 30.18)	0.5568
Percentage body fat	247			247		
≤35.00		Ref.			Ref.	
>35.00 to ≤41.10		10.52 (−33.67, 54.72)	0.6394		25.24 (−19.64, 70.13)	0.2690
>41.10 to ≤45.5		−35.02 (−79.76–9.72)	0.1244		−18.00 (−64.95, 28.96)	0.4510
>45.50		−31.23 (−75.78, 13.33)	0.1687		−7.43 (−55.32, 40.46)	0.7602
Fat mass, kg	247			236		
≤22.70		Ref.			Ref.	
>22.70 to ≤30.80		−0.69 (−44.98, 43.60)	0.9754		9.78 (−34.90, 54.46)	0.6667
>30.80 to ≤39.20		−39.79 (−84.27, 4.68)	0.0792		−25.42 (−71.33, 20.49)	0.2764
>39.20		−44.41 (−88.88, 0.07)	0.0503		−25.30 (−71.82, 21.23)	0.2851
Fat mass index, kg/m^2^	247			236		
≤8.74		Ref.			Ref.	
>8.74 to ≤11.64		13.68 (−30.49, 57.84)	0.5425		26.85 (−17.74, 71.43)	0.2367
>11.64 to ≤14.75		−50.06 (−94.22, −5.89)	0.0265		−36.99 (−82.30, 8.31)	0.1090
>14.75		−34.47 (−78.45, 9.52)	0.1240		−13.37 (−59.52, 32.78)	0.5686
Percentage trunk fat	247			236		
≤32.60		Ref.			Ref.	
>32.60 to ≤39.10		−19.00 (−63.82, 25.82)	0.4046		−7.11 (−52.70, 38.47)	0.7588
>39.10 to ≤44.40		2.80 (−42.02, 47.62)	0.9022		18.30 (−27.81, 64.41)	0.4350
>44.40		−31.24 (−75.87, 13.40)	0.1693		−9.96 (−58.02, 38.09)	0.6832
Trunk fat mass, kg	247			236		
≤12.00		Ref.			Ref.	
>12.00 to ≤16.40		−13.10 (−57.31, 31.10)	0.5599		2.96 (−42.34, 48.26)	0.8977
>16.40 to ≤21.00		−17.27 (−62.39, 27.86)	0.4518		−2.30 (−49.14, 44.53)	0.9229
>21.00		−43.45 (−88.19, 1.30)	0.0570		−24.20 (−71.44, 23.05)	0.3140

BMI indicates body mass index; CI, confidence interval. ^a^ Multivariable analyses were adjusted for age (<50, 50–59, 60–69, ≥70 years), menopause status (no, yes), race (White, all others), and educational level (grade school/some high school/high school graduate or equivalent, some college, college graduate, advanced degree). Urinary creatinine-correction = covariate-adjusted standardization.

**Table 5:** Associations of body size and body composition with creatinine-corrected triclosan stratified by menopausal status.

Variable	Premenopausal ^a^			Postmenopausal ^a^		
	*n*	Beta Estimate (95% CI)	*p*-Value	*n*	Beta Estimate (95% CI)	*p*-Value
BMI, kg/m^2^	106			185		
<25		Ref.			Ref.	
25 to <30		19.06 (−49.58, 87.70)	0.5829		−43.85 (−89.25, 1.56)	0.0583
≥30		−10.55 (−85.06, 63.97)	0.7794		−66.57 (−109.18, −23.96)	0.0024
P-interaction = 0.2197						
Waist circumference, inches	95			163		
≤79.00		Ref.			Ref.	
>79.00 to ≤89.00		60.69 (−12.25, 133.64)	0.1017		−10.24 (−63.82, 43.34)	0.7063
>89.00 to ≤101.40		−74.72 (−170.71, 21.26)	0.1254		−40.48 (−90.36, 9.39)	0.1109
>101.40		−13.56 (−110.47, 83.34)	0.7815		−35.83 (−85.65, 13.99)	0.1573
P-interaction = 0.1615						
Waist-to-hip ratio	95			162		
≤0.78		Ref.			Ref.	
>0.78 to ≤0.83		−76.03 (−165.37, 13.32)	0.0943		11.38 (−34.81, 57.58)	0.6271
>0.83 to ≤0.87		−72.60 (−154.91, 9.71)	0.0831		31.10 (−17.82, 80.03)	0.2110
>0.87		11.87 (−81.58, 105.33)	0.8012		−4.68 (−50.38, 41.03)	0.8401
P-interaction = 0.0348						
Percentage body fat	91			156		
≤35.00		Ref.			Ref.	
>35.00 to ≤41.10		108.94 (32.17, 185.71)	0.0060		−62.88 (−119.14, −6.61)	0.0288
>41.10 to ≤45.50		10.44 (−88.03, 108.91)	0.8335		−66.78 (−119.44, −14.12)	0.0133
>45.50		19.69 (−81.46, 120.83)	0.6995		−58.40 (−111.89, −4.91)	0.0326
P-interaction = 0.0050						
Fat mass, kg	91			156		
≤22.70		Ref.			Ref.	
>22.70 to ≤30.80		73.33 (−9.72, 156.39)	0.0827		−53.93 (−106.85, −1.01)	0.0458
>30.80 to ≤39.20		47.03 (−51.42, 145.48)	0.3447		−78.33 (−128.11, −28.55)	0.0023
>39.20		−13.96 (−110.44, 82.52)	0.7741		−59.96 (−110.62, −9.30)	0.0207
P-interaction = 0.0175						
Fat mass index, kg/m^2^	91			156		
≤8.74		Ref.			Ref.	
>8.74 to ≤11.64		103.97 (21.17, 186.78)	0.0145		−38.48 (−92.09, 15.13)	0.1582
>11.64 to ≤14.75		−11.32 (−102.52, 79.87)	0.8055		−73.34 (−123.94, −22.74)	0.0048
>14.75		16.76 (−83.21, 116.73)	0.7395		−49.63 (−100.07, 0.80)	0.0537
P-interaction = 0.0464						
Percentage trunk fat	91			156		
≤32.60		Ref.			Ref.	
>32.60 to ≤39.10		41.91 (−38.20, 122.02)	0.3010		−72.86 (−127.71, −18.00)	0.0096
>39.10 to ≤44.40		123.17 (37.52, 208.82)	0.0054		−70.73 (−125.08, −16.39)	0.0111
>44.40		−6.46 (−118.06, 105.15)	0.9087		−60.22 (−112.24, −8.20)	0.0236
P-interaction = 0.0011						
Trunk fat mass, kg	91			156		
≤12.00		Ref.			Ref.	
>12.00 to ≤16.40		65.89 (−14.54, 146.32)	0.1070		−73.21 (−127.65, −18.77)	0.0087
>16.40 to ≤21.00		105.43 (2.46, 208.40)	0.0449		−74.91 (−126.84, −22.98)	0.0050
>21.00		−5.38 (−102.26, 91.49)	0.9122		−75.52 (−127.91, −23.12)	0.0050
P-interaction = 0.0037						

BMI indicates body mass index; CI, confidence interval; Ref., reference. ^a^ Multivariable analyses were adjusted for age (<50, 50−59, 60−69, ≥70 years), race (White, all others), and educational level (grade school/some high school/high school graduate or equivalent, some college, college graduate, advanced degree). Urinary creatinine-correction = covariate-adjusted standardization.

## Data Availability

The data set for this study is available from the corresponding author on reasonable request.

## Abbreviations

ABC	Women’s Health After Breast Cancer Study
BPA	Bisphenol A
BMI	Body mass index
DBBR	Data Bank and BioRepository
EDC	Endocrine-disrupting compound
NHANES	National Health and Nutrition Examination Survey
TBBA	Tetrabromobenzoic acid
TBBPA	Tetrabromobisphenol A
WC	Waist circumference
